# Erythritol-Enriched Air-Polishing Powder for the Surgical Treatment of Peri-Implantitis

**DOI:** 10.1155/2015/802310

**Published:** 2015-05-06

**Authors:** Silvio Taschieri, Roberto Weinstein, Massimo Del Fabbro, Stefano Corbella

**Affiliations:** ^1^Department of Biomedical, Surgical and Dental Sciences, Università degli Studi di Milano, Research Centre in Oral Health, IRCCS Istituto Ortopedico Galeazzi, Via Riccardo Galeazzi 4, 20161 Milan, Italy; ^2^Department of Biomedical, Surgical and Dental Sciences, Università degli Studi di Milano, IRCCS Istituto Ortopedico Galeazzi, Via Riccardo Galeazzi 4, 20161 Milan, Italy

## Abstract

Peri-implantitis represents a major complication that can compromise the success and survival of implant-supported rehabilitations. Both surgical and nonsurgical treatment protocols were proposed to improve clinical parameters and to treat implants affected by peri-implantitis. A systematic review of the literature was performed on electronic databases. The use of air-polishing powder in surgical treatment of peri-implantitis was investigated. A total of five articles, of different study designs, were included in the review. A meta-analysis could not be performed. The data from included studies reported a substantial benefit of the use of air-polishing powders for the decontamination of implant surface in surgical protocols. A case report of guided bone regeneration in sites with implants affected by peri-implantitis was presented. Surgical treatment of peri-implantitis, though demanding and not supported by a wide scientific literature, could be considered a viable treatment option if an adequate decontamination of infected surfaces could be obtained.

## 1. Introduction

Dental implants could nowadays be considered a viable treatment option for total or partial edentulism and their use is well supported by the scientific literature [1–3].

Long-term success and survival of implant-supported restorations depend on a number of factors related both to the subjects and to the characteristics of the implant-prosthesis structure [4–7].

Biological and technical complications may occur after implant treatment either immediately after implant or prosthesis placement or after years of prosthetic loading.

Technical complications might not affect the success of the restoration because they can be treated successfully in almost all cases without causing the loss of implant osseointegration [8–10].

Short-term biological complications are usually related to acute infection occurring immediately after surgical intervention or during the healing phase and could cause a massive bone loss, jeopardizing the osseointegration process [11].

Long-term biological complications are related to the inflammatory status of peri-implant tissues. Peri-implant mucositis is a reversible infectious disease characterized by an inflammatory reaction of peri-implant soft tissues with swelling and bleeding on probing but without any relevant bone resorption [12]. If not adequately treated, peri-implant disease can evolve in peri-implantitis that is characterized by bone resorption induced by microbial infection that can lead to the loss of osseointegration [12].

The epidemiology of peri-implant diseases reflects the spreading of implant treatment that occurred over the years. The incidence of peri-implant mucositis ranged from 50% to 90% of implants after 8–10 years [13, 14] while peri-implantitis has been described to affect up to 36.6% of implants [15]. A recent systematic review of the literature confirmed these results, reporting long-term data [16].

Since peri-implant mucositis can be treated successfully removing dental plaque from implant and prosthetic components, with or without the adjunctive aid of antimicrobial agents [17–19], peri-implantitis requires more sophisticated treatment plans.

Nonsurgical treatments applied to peri-implantitis involving the use of Er:YAG laser, chlorhexidine [20, 21], and other devices [22] showing that such treatments can improve clinical parameters without arresting the progression of peri-implantitis. Adding local or systemic administration of antibiotics is possible to obtain bleeding and probing depth reduction, failing in resolving inflammatory lesions in cases of bone loss [23, 24].

Surgical treatment of peri-implantitis aims at arresting the progression of the pathology either filling the bone defect created by the peri-implant infection or eliminating the peri-implant pocket through resective procedures [25]. The decontamination of implant surface before guided bone regeneration (GBR) procedures has to be considered fundamental for achieving a successful integration of the grafting material [25, 26].

The aim of the present paper was to systematically review the existing literature about the use of air-polishing devices and powder for the treatment of peri-implantitis. Moreover a case report was presented.

## 2. Literature Review

### 2.1. Materials and Methods

The search of the literature was performed interrogating electronic databases (Embase, Medline through PubMed interface, and the entire Cochrane Library) using an ad hoc prepared search string including the keywords “air polishing,” “air abrasive,” “glycine,” “peri-implant disease,” and “peri-implantitis” combined with the use of Boolean operators “AND” and “OR.” Relevant articles were also manually selected through the investigation of the reference lists of the included papers.

Inclusion criteria for the studies were (1) studies concerning the use of air-abrasive powder devices for the treatment of implant surfaces in the presence of peri-implantitis; (2) studies describing surgical treatments; (3) any study design.

Two authors (Stefano Corbella and Silvio Taschieri) independently screened abstracts and full texts of the eligible papers for possible inclusion. In case of disagreement in the decision, this was resolved by discussion.

A previously designed electronic sheet served for data recording after extraction.

The primary outcomes that were evaluated were (1) implant survival; (2) changes in clinical parameters as peri-implant probing depth, bleeding, and plaque indexes; (3) percentage of bone fill of the defect for studies about surgical treatment of peri-implantitis.

The secondary outcomes were (1) parameters investigating patients' quality of life or appreciation of the treatment and (2) implant success rate.

Data extracted from comparative studies were included in the meta-analysis that was performed using Review Manager 5.1 (Cochrane Library, http://ims.cochrane.org/revman). The use of air-powder devices was compared to controls. The meta-analysis was carried out using inverse variance method and random effects comparing weighted mean difference.

When data could not be included in a meta-analysis a narrative description was provided.

## 3. Results

The initial research retrieved a total of 127 titles. After title and abstract screening, 13 articles were selected for full-texts examination. Finally, five articles were included in the review [27–31].

Three articles reported results from prospective studies [27–29], one was from a retrospective study [31], and one was a case series [30].

Studies were not comparable in terms of study designs, outcomes, and treatments provided, so a meta-analysis could not be performed.

A summary of included studies was shown in Table 1.

In the study published in 2000, Behneke and coworkers reported data on 25 implants in 17 patients with signs and symptoms of peri-implantitis [27]. Access flap surgery and debridement of the granulation tissue were performed. Then, air-polishing with bicarbonate powder was applied for implant surface decontamination. Finally, guided bone regeneration (GBR) with autogenous bone was used to fill the bony defect. After three years of follow-up, two failures were reported. In the other cases an improvement in all clinical parameters could be observed.

The case series published in 2009 presented the results of surgical treatment of peri-implantitis in 10 implants in 10 patients [28]. After open flap access to the lesion, sodium carbonate air-powder was used to decontaminate implant surface, further debrided with the use of resin curettes. No bone graft was placed. An improvement in clinical parameters was reported in all cases. Moreover, the total amount of TNF-a significantly reduced over time.

The study by Máximo and coworkers, published in 2009, reported results of surgical treatment of peri-implantitis in 13 implants in 13 subjects. The treatment consisted in surgical access flap and debridement of implant surface with Teflon curetted and bicarbonate powder air-abrasive. The authors reported a significant reduction of a number of bacterial species detectable in the pocket sulcus [30].

One prospective study on 20 implants with peri-implantitis in 20 subjects described the clinical outcomes of surgical treatment with open flap and debridement of implant surface with Teflon curettes and bicarbonate air-abrasive powder [29]. At 3 months, all clinical parameters improved as the presence of inflammatory cytokines reduced.

Recently the retrospective study published by Toma and colleagues reported data about the treatment of peri-implantitis through access flap and decontamination with glycine powder air-polishing without application of any bone graft [31]. Twenty-two implants in 22 subjects were treated. Gingival index and probing depth significantly improved in cases treated with glycine powder.

## 4. Case Report

A woman, aged 58, nonsmoker, without any systemic disease (ASA-1 following the classification of the American Society of Anesthesiologists) that may increase the risk of peri-implant or periodontal diseases (i.e., diabetes or immunological impairment) presented with the symptoms of peri-implantitis and was referred to the implant-supported rehabilitation of 2.5 and 2.6. Contextually 2.4 tooth showed a +2 mobility.

Implants were placed in female, 64 years before the patient came to the attention of the authors. The subject referred to pain and swelling in the region of 2.5 and 2.6, associated with sporadic episodes of alithosis. The clinical assessment allowed finding a probing depth of 14 mm mesial/circumferentially to 2.5 with bleeding. After bidimensional radiographic assessment (with periapical and panoramic radiograph) a concave bone resorption can be observed of about 11 mm mesial and distal to both implants. The radiographic images also showed a root-end resection involving 2.4 tooth with a periapical large lesion encroached with the peri-implant disease above mentioned.

The implant-supported rehabilitation of 2.5 and 2.6 was separated in order to achieve a complete clinical diagnosis of the mobility of both implants. 2.5 did not show any mobility, while 2.6 showed a mobility of more than 1 mm.

The patient was informed about her critical clinical conditions. One treatment alternative was proposed which would have implied the removal of both implants and 2.4, the tridimensional bone reconstruction of the area, and implant placement after complete healing of the bone grafting procedure.

The subject, informed about advantages and disadvantages of the proposed alternative, refused, asking for an option that would have avoided major bone grafting procedure, maintaining the implants in site if possible.

### 4.1. Surgical Procedure

**Figure 1:**
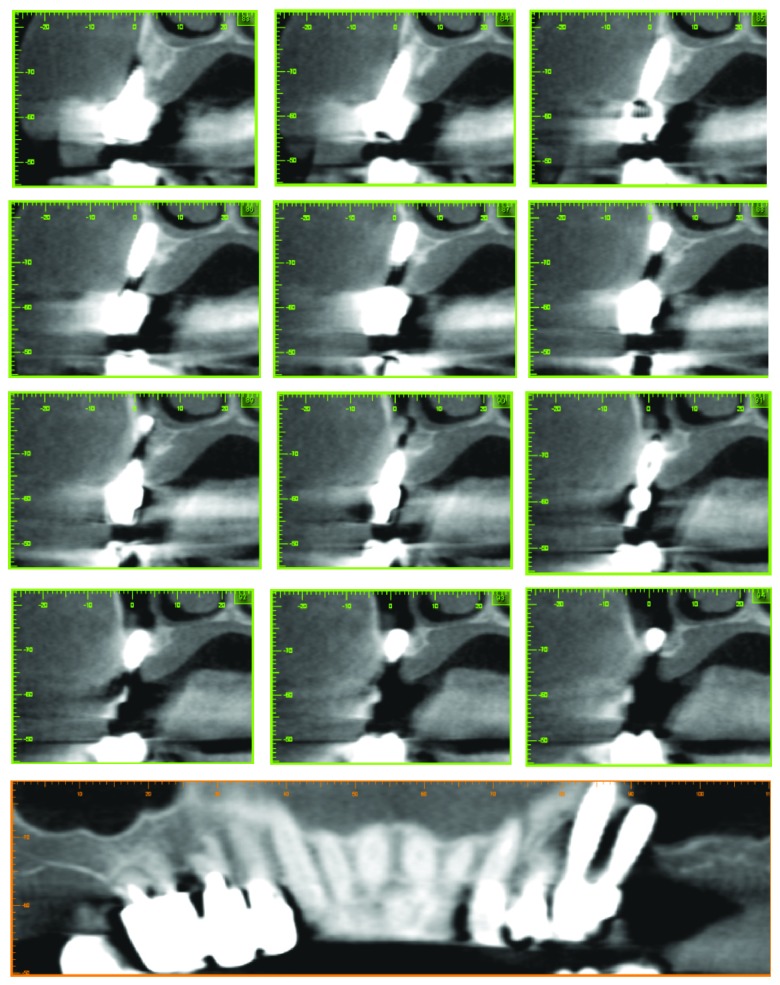
CBCT scans images showing the large concave bone resorption due to peri-implant diseases involving implant-supported rehabilitation of 2.5 and 2.6.

After an appropriate planning through tridimensional imaging from CBCT scans (Figure 1), a surgical approach with GBR of 2.5 together with the extraction of 2.4 and 2.6 (that was considered hopeless due to massive bone resorption and active infection) was decided. It was planned, after adequate healing, to place a fixed denture with mesial cantilever of a single tooth on 2.5.

After complete acceptation of the proposed option, the patient signed an informed consent form.

The surgical intervention was performed by a clinician with more than ten years of experience in oral surgery (ST).

After local anesthesia with articaine 4% and epinephrine 1 : 100.000 a full-thickness flap was elevated, extending from 2.3 to the distal portion of 2.6 with a distal vertical release incision. The tooth 2.4 and the implant in position 2.6 were extracted because of massive bone resorption and mobility. The granulation and infected tissue were accurately removed using surgical curettes. Implant surface was debrided and decontaminated using an air-powder device using a powder consisting of erythritol, amorphous silica, and 0.3% chlorhexidine (Air-Flow Plus, E.M.S. Electro Medical Systems, Nyon, Switzerland). The air-powder device was used circumferentially for about 3 minutes at a distance of less than 1 cm from implant surface.

**Figure 2:**
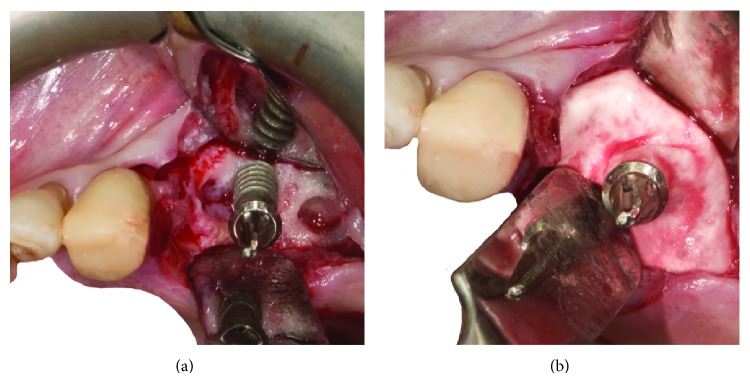
(a) Surgical site after extraction of 2.6 implant and 2.4 tooth contextually to accurately remove granulation and infected tissue. An intact portion of Schneiderian membrane is visible in the 2.6 implant site. (b) Surgical site showing the GBR technique used. A xenogeneic scaffold covered by a resorbable membrane was positioned circumferentially around the 2.5 implant generating a “regeneration chamber.”

After the treatment a bone substitute (deproteinized bovine bone matrix-Bio-Oss, Geistlich Pharma AG, Wolhusen, Switzerland) mixed with pure platelet-rich plasma (P-PRP [32]), prepared following the protocol described in previously published reports [33], was used as grafting material around the 2.5 implant. Two resorbable collagen membranes (Bio-Gide, Geistlich Pharma AG, Wolhusen, Switzerland) were then used. One was placed in order to isolate the communication with the preserved Schneiderian membrane due to the extraction of 2.6 implant and the other to cover the graft (Figure 2).

The flap was then mobilized, releasing muscular insertion in order to allow a primary closure. The flap was finally closed with interrupted sutures made of nonresorbable 5/0 ETHILON (Ethicon Inc., Blue Ash, OH, USA) and three sutures were horizontal mattress (5/0 VICRYL, Ethicon Inc., Blue Ash, OH, USA) in a paramarginal position to increase flap stability during the healing phase. The patient was advised to avoid mouth rinsing, hard and hot food, hot drinks, heavy physical work, and tooth brushing during the day of surgery. Ice packs were provided after surgery. Moreover the patient was instructed to rinse their mouth twice daily with chlorhexidine digluconate 0.2% for plaque control up to 7 days after surgery. The subject was prescribed nonsteroidal analgesics after the surgical procedure for pain relief and/or swelling control if needed. Antibiotic therapy was also prescribed (amoxicillin 1 g, three times a day for five days). Sutures were removed after 7 days.

### 4.2. Clinical and Radiographic Evaluation

No complications occurred in the postsurgical period. The patient reported a moderate pain on the first two days after surgery with little swelling and without hematoma. No dehiscence of the flap occurred.

**Figure 3:**
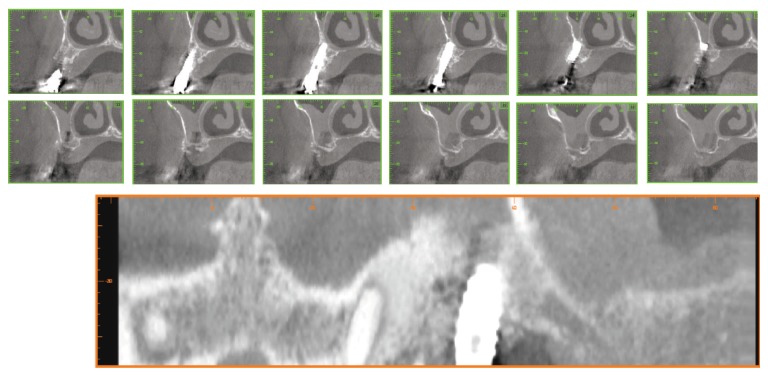
CBCT scans images after six months from surgical intervention showing a substantial stability of bone graft that allowed hypothesizing of the reosseointegration of the implant affected by peri-implantitis.

After six months from surgical intervention a cone-beam computed tomography served to evaluate the stability of tridimensional bone graft over time (Figure 3). Both clinical and radiographic examination confirmed a substantial stability of bone graft that allowed hypothesizing the reosseointegration of the implant affected by peri-implantitis.

**Figure 4:**
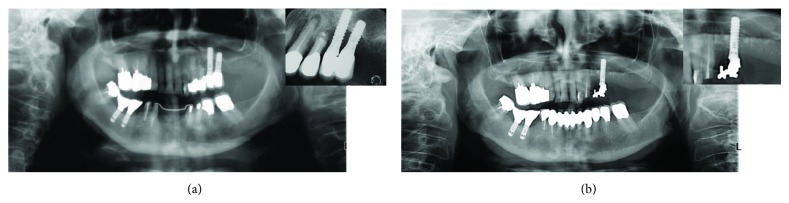
Comparison between periapical and panoramic radiographs before and after 12 months from surgery.

Figure 4 showed periapical and panoramic radiographic images 12 months after surgical operation confirming the above mentioned six-month refers.

## 5. Discussion

The present paper reported the results of a narrative review about the use of air-powder device in the surgical treatment of peri-implantitis. The presented case report showed that when an accurate decontamination of implant surface is performed the reosseointegration of affected implants could be achieved even though a longer follow-up is mandatory to confirm these preliminary observations.

Surgical treatment of peri-implantitis has the objective of arresting the progression of the disease through decontamination of implant surface and removal of the inflammatory tissues [34]. Guided bone regeneration (GBR) procedures could be used in surgical treatment aiming at the reconstruction of the bone volume that was lost due to the infective process. The reosseointegration of the implant surfaces affected by peri-implantitis has to be considered the most important objective when a GBR procedure was performed [34, 35].

Some controlled clinical studies on surgical treatment of peri-implantitis using GBR reported some clinical benefits in comparison to control groups, in which GBR was not performed [36–38].

To obtain a reosseointegration of affected implants and, in general, the success of surgical procedure a decontamination/detoxification of the implant surface has to be considered mandatory [39]. Detoxification techniques could be divided into chemical and mechanical ones on the basis of the action of the agents involved [39]. Chemical decontamination could be performed through the use of saline solutions [40, 41], citric acid [41–43], chlorhexidine [41], hydrogen peroxide [43], and other antimicrobials. Mechanical decontamination implies the action of an agent to physically remove the microorganisms from implant surface. Laser and photodynamic therapy have been proven to be effective in the decontamination of implant surface [44, 45]. Another form of mechanical implant decontamination is implantoplasty through the removal, with burs, of rough (and potentially contaminated) portion of implant surface. The aim of the implantoplasty is to remove the contaminated surfaces and create the conditions to reduce plaque accumulation [46, 47]. Air-powder abrasives were studied in a number of articles which showed that the use of amino acid glycine powder can be effective in removal of bacterial biofilm without altering the morphological characteristics of implant surface [48, 49]. Further, air-abrasive powder has the advantage of preserving the surface characteristics of titanium without creating roughness and alterations that can become a bacterial niche [50].

Guided bone regeneration can be applied to the surgical treatment of peri-implantitis with the aim of filling the bone cavity created by the inflammatory process [34]. Even though a number of articles reported good clinical results, implant surface characteristics and the low contamination appeared to be key factors to permit bone regeneration [19, 51].

The present paper reported that the use of abrasive powder devices can be successfully associated with guided bone regeneration to obtain a significant improvement in clinical parameters. The case report showed that the use of powder enriched with erythritol allowed a debridement of implant surface, without altering its structure, and potentially removing the bacterial biofilm as was proved in a recently published in vitro study on the same powder as compared to glycine [52]. Moreover, guided bone regeneration appeared to be successful in the short time clinically and radiographically without presenting any sign of residual infection, even though it was applied to a large lesion.

In conclusion, surgical treatment of peri-implantitis is described as a viable option to preserve the affected implant and to stabilize the clinical parameters, thus arresting the pathology progression. Guided tissue regeneration could be achieved only after the disruption of bacterial biofilm from implant surface which can be achieved using abrasive powder devices.

Moreover, studies with a wider sample size and a longer follow-up are needed to validate the procedure described in the present paper.

## Figures and Tables

**Table 1:** General characteristics of the included studies.

Authors	Year	Study type	Number of subjects/ implants	Type of defect	Treatment	Considered parameters	Results
Behneke et al. [27]	2000	Pros.	17/25	NR	Surgical debridement and air-polishing with bicarbonate + GBR with autogenous bone	Marginal bone loss (MBL); horizontal bone loss (HBL); vertical bone loss (VBL)	Two failures; median MBL: from 6.3 mm to 2.1 mm (3 y); median HBL: from 1.8 mm to 2.1 mm (3 y); median VBL: from 4.5 mm to 0.0 mm

de Mendonça et al. [28]	2009	Case series	10/10	NR	Surgical debridement + abrasive sodium carbonate air-powder + resin curettes	BI; PI; PD; CAL; TNF-a	Mean PD reduction: 2.4 mm (1 y); mean CAL reduction: 2.0 mm (1 y). The total amount of TNF-a significantly reduced over time

Máximo et al. [30]	2009	Pros.	13/13	NR	Access flap + Teflon curettes + air-powder (sodium carbonate)	PD; CAL	25% with PD ≥ 5 mm after 3 months. Levels of *Treponema denticola*, *Tannerella forsythia,* and *Parvimonas micra* and of *Fusobacterium nucleatum* were lower after treatment

Duarte et al. [29]	2009	Pros.	20/20	NR	Access flap + resin curettes + air-powder (sodium carbonate)	PD	Mean PD: from 7.5 mm to 4.4 mm (3 mo). Significant difference in inflammatory cytokines between healthy and affected implants

Toma et al. [31]	2014	Retro.	22/22		Air-abrasive device versus plastic curettes + cotton pellets + saline; no GBR	PD	Significant reduction of clinical parameters in all groups; better improvements for air-abrasive device regarding gingival index and probing depth; no peri-implantitis resolution

NR: not reported; PD: probing depth; CAL: clinical attachment level.
